# Impact of *γ* factor in the penalty function of Bayesian penalized likelihood reconstruction (Q.Clear) to achieve high-resolution PET images

**DOI:** 10.1186/s40658-023-00527-w

**Published:** 2023-01-22

**Authors:** Kenta Miwa, Tokiya Yoshii, Kei Wagatsuma, Shogo Nezu, Yuto Kamitaka, Tensho Yamao, Rinya Kobayashi, Shohei Fukuda, Yu Yakushiji, Noriaki Miyaji, Kenji Ishii

**Affiliations:** 1 Department of Radiological Sciences, School of Health Sciences, Fukushima Medical University, 10-6 Sakaemachi, Fukushima-shi, Fukushima 960-8516 Japan; 2 Research Team for Neuroimaging, Tokyo Metropolitan Institute of Gerontology, 35-2, Sakae-cho, Itabashi-ku, Tokyo, 173-0015 Japan; 3 Department of Radiology, Fukushima Medical University Hospital, 1 Hikarigaoka, Fukushima, Fukushima 960-1295 Japan; 4 School of Allied Health Sciences, Kitasato University, 1-15-1 Kitazato, Minami-ku, Sagamihara, Kanagawa 252-0373 Japan; 5 Department of Radiology, Ehime University Hospital, 454 Shitsukawa, Touon-shi, Ehime 791-0204 Japan; 6 Department of Radiology, Tokai University Hospital, 143 Shimokasuya, Isehara-shi, Kanagawa 259-1193 Japan; 7 Department of Radiological Sciences, School of Health Sciences, International University of Health and Welfare, 2600-1 Kitakanemaru, Ohtawara, Tochigi 324-8501 Japan; 8 Department of Nuclear Medicine, Cancer Institute Hospital of Japanese Foundation for Cancer Research, 3-8-31 Ariake, Koto-ku, Tokyo, 135-8550 Japan

**Keywords:** *β* value, BPL, BSREM, *γ* factor, Q.Clear, Sub-centimeter lesion

## Abstract

**Background:**

The Bayesian penalized likelihood PET reconstruction (BPL) algorithm, Q.Clear (GE Healthcare), has recently been clinically applied to clinical image reconstruction. The BPL includes a relative difference penalty (RDP) as a penalty function. The *β* value that controls the behavior of RDP determines the global strength of noise suppression, whereas the *γ* factor in RDP controls the degree of edge preservation. The present study aimed to assess the effects of various *γ* factors in RDP on the ability to detect sub-centimeter lesions.

**Methods:**

All PET data were acquired for 10 min using a Discovery MI PET/CT system (GE Healthcare). We used a NEMA IEC body phantom containing spheres with inner diameters of 10, 13, 17, 22, 28 and 37 mm and 4.0, 5.0, 6.2, 7.9, 10 and 13 mm. The target-to-background ratio of the phantom was 4:1, and the background activity concentration was 5.3 kBq/mL. We also evaluated cold spheres containing only non-radioactive water with the same background activity concentration. All images were reconstructed using BPL + time of flight (TOF). The ranges of *β* values and *γ* factors in BPL were 50–600 and 2–20, respectively. We reconstructed PET images using the Duetto toolbox for MATLAB software. We calculated the % hot contrast recovery coefficient (CRC_hot_) of each hot sphere, the cold CRC (CRC_cold_) of each cold sphere, the background variability (BV) and residual lung error (LE). We measured the full width at half maximum (FWHM) of the micro hollow hot spheres ≤ 13 mm to assess spatial resolution on the reconstructed PET images.

**Results:**

The CRC_hot_ and CRC_cold_ for different *β* values and *γ* factors depended on the size of the small spheres. The CRC_hot,_ CRC_cold_ and BV increased along with the *γ* factor. A 6.2-mm hot sphere was obvious in BPL as lower *β* values and higher *γ* factors, whereas *γ* factors ≥ 10 resulted in images with increased background noise. The FWHM became smaller when the *γ* factor increased.

**Conclusion:**

High and low *γ* factors, respectively, preserved the edges of reconstructed PET images and promoted image smoothing. The BPL with a *γ* factor above the default value in Q.Clear (*γ* factor = 2) generated high-resolution PET images, although image noise slightly diverged. Optimizing the *β* value and the *γ* factor in BPL enabled the detection of lesions ≤ 6.2 mm.

## Introduction

^18^F-fluoro-2-deoxy-d-glucose (^18^F-FDG) positron emission tomography/computed tomography (PET/CT) has become an important imaging tool for detecting, localizing, characterizing, staging and determining the therapeutic response of cancer [1]. However, conventional PET images have limitations due to relative low spatial image resolution and a generally poor signal-to-noise ratio (SNR) [2]. Recent advances in PET image reconstruction have improved detectability [3, 4]. Image reconstruction methods and their conditions can improve spatial image resolution and the SNR of ^18^F-FDG PET images [5].

Regularization is desirable for PET image reconstruction, and one of the most powerful regularization techniques for achieving this is the penalized likelihood reconstruction algorithm [6]. Q.Clear (GE Healthcare, Milwaukee, WI, USA) is a Bayesian penalized likelihood reconstruction algorithm that has been clinically applied [7]. This algorithm provides accurately reconstructed images using a penalty function to suppress noise and point spread function (PSF) modeling [8]. The BPL enhances spatial resolution and image quality and augments the standardized uptake value (SUV) of small lesions compared with conventional reconstruction using ordered subset expectation maximization (OSEM) [8–13].

Q.Clear includes a relative difference penalty (RDP) as a penalty function, which is a function of the relative difference between neighboring voxels that avoids excessive smoothing over large edges [14]. The behavior of this penalty function is controlled by a penalization factor (*β* value), which determines the global strength of noise suppression and is the only user-input variable in Q.Clear. We previously found that an optimized *β* value in BPL allowed the detection of micro hollow spheres ≤ 10 mm [15]. Howard et al. reported that a BPL with *β* = 150 was advantageous when evaluating small pulmonary nodules with a diameter of ~ 8 mm [9]. The BPL with a low *β* value can detect sub-centimeter lesions.

The RDP of the penalty function is defined as [16]:

$$R\left( x \right) = \mathop \sum \limits_{j} \mathop \sum \limits_{{k \in N_{j} }} w_{jk} \sqrt {\beta_{j} \beta_{k} } \frac{{\left( {x_{j} - x_{k} } \right)^{2} }}{{x_{j} + x_{k} + \gamma \left| {x_{j} - x_{k} } \right|}},$$

where *N*_*j*_ is a set of voxels neighboring voxel *j*, *w*_*jk*_ is weight depending on the distance between voxels* j* and *k*, and *β*_*j*_ is a penalty modulation factor. The *γ* factor is a penalty parameter that controls the degree of edge preservation. Q.Clear has a fixed *γ* factor of 2 for the RDP [7, 8]. However, the ability of the *γ* factor to detect small lesions has not yet been determined. We postulated that BPL with a higher *γ* factor in the RDP could achieve high-resolution PET images, despite the possibility of apparently unnatural, blocky images [14, 17]. Therefore, the present study aimed to determine the relationship between the *β* value and *γ* factor in the penalty function and to quantify the ability of the *γ* factor in BPL reconstruction to generate high-resolution PET images. To our knowledge, this is the first attempt to investigate the basic characteristics of the *γ* factor of Q.Clear from a user perspective.

## Materials and methods

### PET/CT systems

We acquired PET data using a Discovery MI PET/CT system (GE Healthcare, Milwaukee, WI, USA) with a PET scanner comprising a lutetium-based scintillator (LBS) a silicon photomultiplier (SiPM)-PET and 64-slice CT scanners and a LightBurst digital detector with four axially arranged blocks of detectors contained 19,584 Lutetium Yttrium Orthosilicate (LYSO) scintillator crystals of 3.95 × 5.3 × 25 mm in a 4 × 9 matrix.

### Phantoms

We used a National Electrical Manufacturers Association International Electrotechnical Commission (NEMA IEC) body phantom (9.84 L capacity) containing spheres with inner diameters of 10, 13, 17, 22, 18, 37 mm and 4.0, 5.0, 6.2, 7.9, 10, 13 mm (NEMA IEC Body Phantom Set™ and Micro Hollow Sphere Set, respectively; Data Spectrum Corp., Durham, NC, USA) (4). The target-to-background ratio (TBR) in the phantom was 4:1 with a background activity concentration of 5.3 kBq/mL. We also evaluated a NEMA IEC phantom with six cold spheres (10–37 mm) containing only non-radioactive water on the same background activity concentration.

### Data acquisition and image reconstruction

We acquired three-dimensional PET data for 10 min. All images were reconstructed using BPL + TOF that consisted of 2 iterations of non-TOF OSEM and 3 iterations of the modified block sequential regularized expectation maximization algorithm (BSREM) followed by 8 iterations of BSREM + TOF, all with 28 subsets. The ranges of *β* values and *γ* factors in BPL were 50–600 and 2–20, respectively. Gaussian-filtering was not applied to the BPL images. The matrix size was 384 × 384 pixels with sizes of 1.04 × 1.04 mm. The PET images were reconstructed on a workstation running the PET Duetto reconstruction toolbox (GE Healthcare) for MATLAB R2017b (The MathWorks Inc., Natick, MA, USA) available from GE Healthcare through a research collaboration agreement.

### Image analyses

The relationship between the *β* value and the *γ* factor in BPL was revealed by the results generated by standard NEMA body phantom. We then investigated phantoms containing sub-centimeter spheres were subsequently investigated. All PET datasets were analyzed using PMOD ver. 3.8 software (PMOD Technologies LLC, Zurich, Switzerland).

A circular region of interest (ROI) with the same diameter as a hot sphere was placed on the central slice of each hot sphere under CT imaging guidance. Twelve circular ROIs (diameter, 10 mm) were placed on central slices of each sphere and on slices located ± 1 and ± 2 cm distant from the central slice (total, 60 ROI) as background ROI [15]. The activity concentrations in the all ROIs were recorded. We then calculated the % hot contrast recovery coefficient (CRC_hot_) of the hot spheres, the cold CRC (CRC_cold_) for the cold spheres [18], background variability (BV) and residual lung error (LE) [19] as follows:

1 $${\text{CRC}}_{{{\text{hot}}}} \left( {\text{\% }} \right) = \left( {\frac{{{\text{AC}}_{{{\text{hot}}}} /{\text{AC}}_{{{\text{BG}}}} - 1}}{{A_{{\text{H}}} /A_{{\text{B}}} - 1}}} \right) \times 100$$

2 $${\text{CRC}}_{{{\text{cold}}}} \left( \% \right) = \left( {{1} - \left[ {{\text{AC}}_{{{\text{cold}}}} /{\text{AC}}_{{{\text{BG}}}} } \right]} \right) \times {1}00$$

3 $${\text{BV}} \left( \% \right) = \frac{{{\text{SD}}_{{{\text{B}}, 10\;{\text{mm}}}} }}{{{\text{AC}}_{{{\text{BG}}, 10\;{\text{mm}}}} }} \times 100$$

4 $${\text{LE}} \left( \% \right) = \frac{{{\text{AC}}_{{{\text{lung}}}} }}{{{\text{AC}}_{{{\text{BG}}, 37\;{\text{mm}}}} }} \times 100,$$

where AC_hot_ is the mean measured activity concentration (Bq/mL) of the each hot sphere ROI, AC_BG_ is the average measured activity concentration of the background calculated from the circular ROIs (*n* = 12) of each sphere size, *A*_H_ is the activity concentration in the hot sphere (Bq/mL), and *A*_B_ is the background activity concentration. AC_cold_ is the mean measured activity concentration (Bq/mL) in each cold sphere ROI. SD_B,10 mm_ is the SD of the activity concentration in the background ROI for a hot sphere with a diameter of 10 mm, and AC_BG,10 mm_ is the average activity concentration of the background calculated from each circular ROI (*n* = 12). AC_lung_ is the mean measured activity concentration in a circular ROI with a diameter of 30 mm drawn down the middle of the lung insert in the central slice, and AC_BG,37 mm_ is the average activity concentration of 12 background ROIs with a diameter of 37 mm.

We drew profile curves on the micro-hollow hot spheres ≤ 13 mm and measured the full width at half maximum (FWHM) to assess the spatial resolution of reconstructed PET images.

## Results

**Fig. 1:**
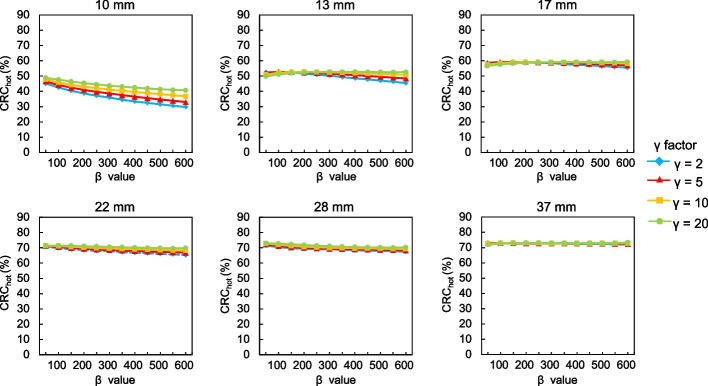
Percent hot contrast recovery coefficient (CRC_hot_) of hot spheres as function of *β* 50–600 for each *γ* factor

**Fig. 2:**
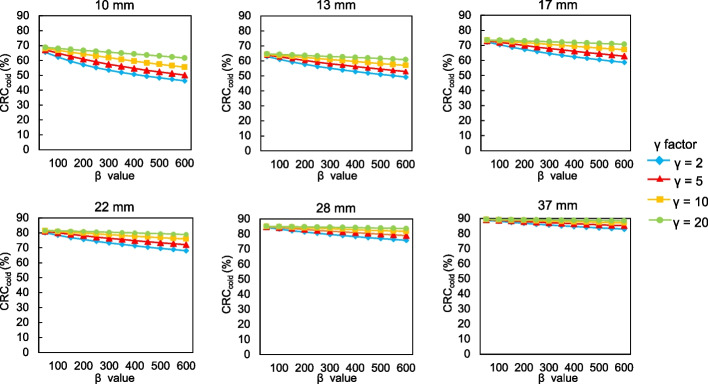
Percent cold contrast recovery coefficients (CRC_cold_) of cold spheres as function of *β* 50–600 for each *γ* factor

**Fig. 3:**
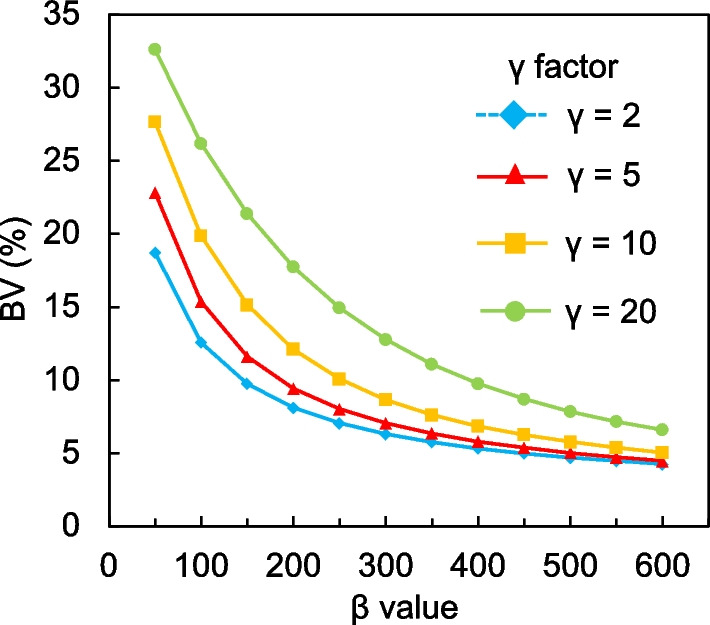
Background variability as a function of *β* 50–600 for each *γ* factor

**Fig. 4:**
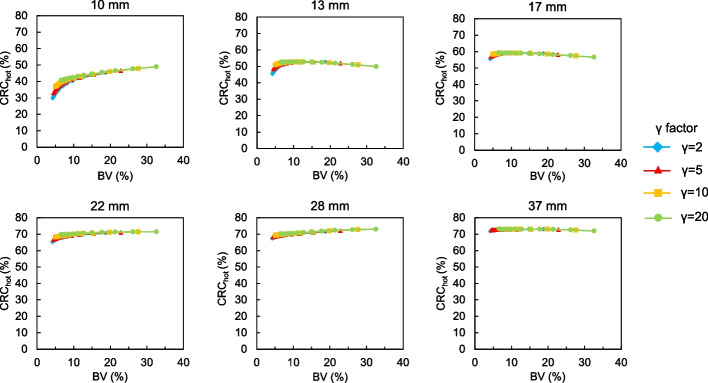
Relationship between CRC_hot_ and BV curves of hot spheres with diameters of 10, 13, 17, 22, 28 and 37 mm using BPL reconstructions with different *γ* factors. Curves for BPL run from left to right as *β* values decrease

Figures 1 and 2 show CRC_hot_ and CRC_cold_, respectively, as a function of the *β* values for each *γ* factor. The trends of CRC_hot_ and CRC_cold_ were similar. The CRC_hot_ and CRC_cold_ increased as a function of decreasing *β* values. The CRC_hot_ for different *β* values and *γ* factors depended on sphere sizes ≤ 13 mm. The CRC_hot_ and CRC_cold_ were less affected by differences in the *β* values and remained stable as the *γ* factor increased, and CRC_cold_ was particularly remarkable. Figure 3 shows the BV of 10 mm spheres as a function of the *β* value for each *γ* factor. The BV decreased as the *β* value increased. In contrast, the BV increased along with the *γ* factor for all *β* values. Figure 4 shows the relationship between the CRC_hot_ and BV of hot spheres with diameters of 10, 13, 17, 22, 28 and 37 mm using BPL reconstructions with each *γ* factor. The curves of CRC_hot_ and BV were almost independent of the *γ* factor for spheres ≥ 17 mm. As the *β* value increased in 10-mm spheres, CRC_hot_ and the BV decreased for all *γ* factors.

**Fig. 5:**
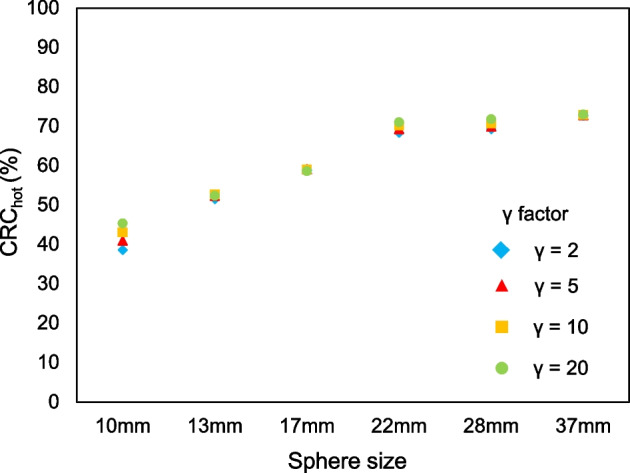
Percent hot contrast recovery coefficient as a function of sphere size with different *γ* factors in images reconstructed using BPL and *β* = 200

Figure 5 shows CRC_hot_ as a function of sphere size with different *γ* factors in BPL images reconstructed using a *β* value of 200. The CRC_hot_ in all *γ* factors improved as the sphere sizes increased. The CRC_hot_ among *γ* factors were compatible. Table 1 shows the residual LE (%) for different *β* values and *γ* factors. The LE of BPL had low (range 4.3‒5.3%) and similar values for *β* and *γ* factors. The LE slightly increased as the *β* value and *γ* factor, respectively, increased and decreased.

**Table 1:** Residual lung effects (LE %) of different *β* values and *γ* factors

*β* value	*γ* factor			
	2	5	10	20
B50*	4.4	4.3	4.3	4.3
B100	4.5	4.4	4.4	4.3
B150	4.6	4.5	4.4	4.3
B200	4.7	4.6	4.5	4.4
B250	4.8	4.7	4.5	4.4
B300	4.8	4.7	4.6	4.5
B350	4.9	4.7	4.6	4.5
B400	5.0	4.8	4.7	4.5
B450	5.0	4.9	4.7	4.6
B500	5.1	4.9	4.8	4.6
B550	5.2	4.9	4.8	4.6
B600	5.3	5.0	4.8	4.7

^*^B*n* indicates BPL reconstruction with *β* value of *n*

**Fig. 6:**
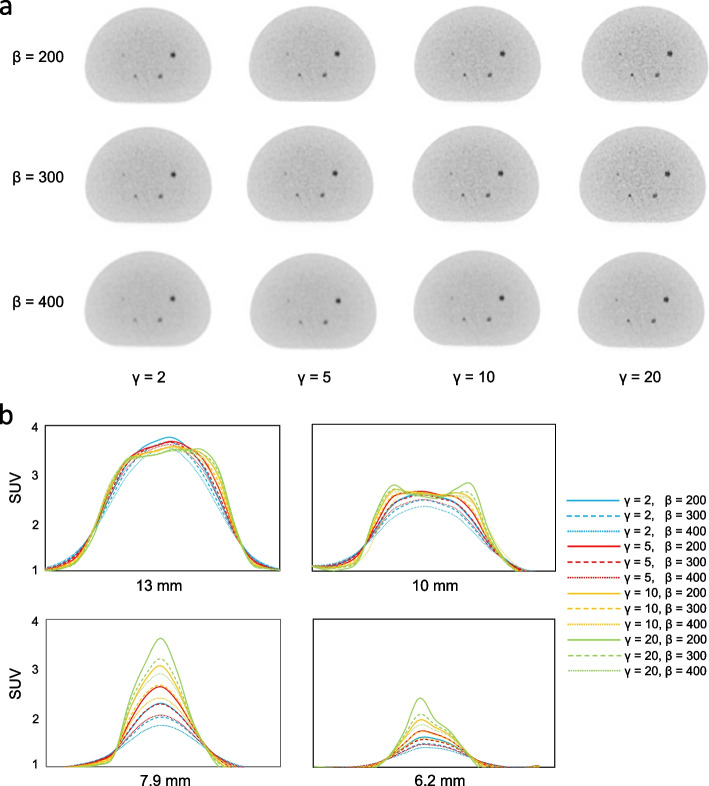
Representative PET images reconstructed using various *β* values and *γ* factors (**a**) and profile curves of spheres (**b**). All images are shown as standard uptake values on a scale from 0 to 4

**Fig. 7:**
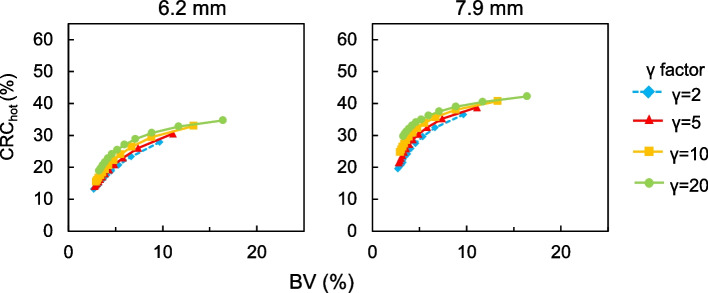
Relationships between CRC_hot_ and BV curves of hot spheres with inner diameters of 6.2 and 7.9 mm determined using BPL reconstruction with various *γ* factors. Values for BPL curve run from left to right as *β* values decrease

**Fig. 8:**
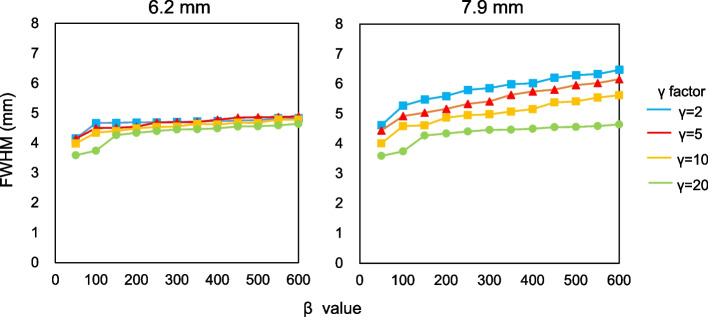
Full width at half maximum determined by profile curves of 6.2- and 7.9-mm hot spheres as function of *β* 50–600 for each *γ* factor

Figure 6 shows representative axial images reconstructed using various *β* values and *γ* factors in the phantom study of the sub-centimeter spheres and their profile curves of each spheres. The 6.2-mm hot sphere was clearly recognized at a lower *β* value and a higher *γ* factor in images reconstructed using BPL, whereas a *γ* factor ≥ 10 resulted in increased background noise on images. Figure 7 shows the relationship between CRC_hot_ and BV of hot spheres with inner diameters of 6.2 and 7.9 mm determined using BPL reconstruction with each *γ* factor. As the *β* value increased, the CRC_hot_ and BV of all *γ* factors decreased. The CRC_hot_ was better at comparable noise levels when the *γ* factor was > 2 than fixed at 2 (the default value in Q.Clear). Figure 8 shows the FWHM determined from a profile curve of hot spheres as a function of the *β* value (50–600) for each *γ* factor. The FWHM considerably improved when the *β* value decreased and the *γ* factor increased.

## Discussion

We evaluated the relationships between *β* values and *γ* factors in BPL reconstruction using a NEMA IEC body phantom and assessed the effects of varying *γ* factors in RDP on the ability to detect sub-centimeter hot spheres. We found that a *γ* factor above the default value of 2 in Q.Clear was appropriate and that optimizing these parameters in BPL enabled the detection of sub-centimeter lesions.

The CRC_hot_ and CRC_cold_ increased as *β* values and *γ* factors, respectively, decreased and increased, but depended on the size of the small spheres (Figs. 1, 2). This might have been due to higher spatial resolution when the *β* value was low and/or the *γ* factor is high. The RDP in BPL reconstruction combines activity-dependent quadratic smoothing with a controlled level of edge preservation [17]. Activity-dependent quadratic smoothing ensures that regions of low activity have a larger penalty and are smoother. This property smoothed out sub-centimeter hot spheres with low activity as a function of increasing *β* values. Since the potential can blur lesions into the background, the *β* value in BPL should be cautiously applied to smaller lesions [20]. In addition, the spatial resolution reconstructed with BPL is better when the *β* value is low. This exerts positive effects on the CRC_hot_ and CRC_cold_ of small spheres [12, 15]. In contrast, higher *γ* factors provide better contrast recovery and more accurate quantitation due to improved edge preservation. However, this can be accompanied by undesirably higher noise levels (Figs. 3, 4). We therefore believe that improving contrast using a higher *γ* factor and controlling concomitant image noise using an adequate *β* value is more effective.

The CRC_hot_ values among *γ* factors were almost identical (Fig. 5). The BPL, namely Q.Clear, can reach full convergence of image quantitation while suppressing noise using the penalty function [5, 16] and improves the penalty function by suppressing the amplitude of a limiting cycle using BSREM [15, 21–23]. Q.Clear also gives significantly better cold contrast recovery than OSEM reconstruction [24]. Thus, these characteristics of Q.Clear are considered to guarantee improved contrast and quantitation regardless of different *γ* factors. Slight differences among CRC_hot_ were caused by the spatial resolution becoming higher as the *γ* factor increased. The LE of BPL remained low (range 4.3%‒5.3%) for all *β* values and *γ* factors (Table 1). This indicated that corrections using BPL reconstruction were accurate [25]. The LE slightly increased as the *β* value and *γ* factor, respectively, increased and decreased [8, 26]. This might have been caused by spillover from the hot uniform area to the lung cold region as a result of smoothing under conditions of higher *β* values and/or lower *γ* factors.

The developers of Q.Clear empirically selected a *γ* factor of 2 as a reasonable trade-off between visual image quality and lesion quantitative accuracy [7, 14]. However, this default value was determined to detect lesions ≥ 10 mm because the supposed detection limit for other PET/CT scanners is ~ 1 cm [27]. The recent replacement of photomultiplier tubes (PMT) with SiPM has led to PET detectors with smaller crystals, better timing resolution and higher photon-detection efficiency [28]. A SiPM-PET/CT system combined with BPL reconstruction can detect of lesions < 1 cm [15, 29, 30]. Thus, the optimal *γ* factor of BPL in a SiPM-PET/CT scanner should be determined to detect sub-centimeter lesions as described herein. Enhanced BPL capability is critical, especially in oncology where identifying early metastases < 1 cm is essential to selecting optimal patient management strategies [4].

The 6.2-mm hot sphere was recognizable with BPL using higher *γ* factors (Fig. 6). This is mainly due to the effects of the RDP on BPL. The RDP with intermediate values for the *γ* factor operates in two modes [31]. A lower *γ* factor in RDP works as a quadratic mode for smoothing, whereas a higher *γ* factor works as a linear mode for edge-preserving. Thus, we consider that the edge-preserving effect of the linear function is superior to smoothing under conditions of higher *γ* values when the BPL yielded a higher contrast for sub-centimeter spheres. Asma et al. reported that a higher *γ* factor provided more accurate quantitation capacity (measured as contrast and recovery coefficient) due to improved spatial resolution [17]. Ahn et al. also described that as the parameter *γ* increases, the RDP approaches the total variation penalty, which yields sharper edges, resulting in less bias in quantitation and less partial volume errors, particularly for small lesions [7].

Contrast and image noise in PET images increased together with the *γ* factor (Fig. 7). This result was consistent with the findings of the Q.Clear developers [7, 14]. A balance between the high contrast and low image noise is needed to determine optimal *β* values and *γ* factors [32, 33]. These points should preferably be located in the top left of the plot [8, 32]. The balance between CRC_hot_ and BV improved as the *γ* factor increased. The better contrast/noise trade-offs were *β* = 200, 250 and 300 when *γ* factors were 5, 10 and 20, respectively. Setting a *γ* factor > 2 is appropriate for detecting sub-centimeter lesions. However, very large *γ* factors produce blocky images [14, 17, 31]. Here, we did not have such visual problems even on PET images with *γ* factors > 5, although image noise was slightly diverged. The FWHM became smaller when the *γ* factor increased (Fig. 8). Optimization of a higher *γ* factor in RDP improved the FWHM and spatial resolution. These results facilitated the detection of sub-centimeter lesions by varying *γ* factors in RDP.

The present study has several limitations. We generated data based on a phantom that represented a patient with small lesions. Patients with tumor states such as transitioning and lymph node metastases should be investigated. The optimal *β* value and *γ* factor in BPL for detecting lesions < 1 cm might differ among PET/CT scanners. Therefore, each PET system should be considered before the present findings could be applied to other image acquisition conditions. Furthermore, the optimal parameter values that we found were valid only for the level of noise and the applied concentration ratio. Tumor and background activity concentrations might be quite variable even within individual patients.

## Conclusions

We investigated the effects of different *γ* factor values for the RDP in BPL reconstruction on the ability to detect sub-centimeter hot spheres in PET images. High and low *γ* factors, respectively, preserved the edges of reconstructed PET images and promoted image smoothing. The BPL with a *γ* > 2 provides high-resolution PET images, although image noise slightly diverged. Optimization of the *β* value and *γ* factor in BPL enable the detection of lesions ≤ 6 mm.

## Data Availability

The datasets used and/or analyzed during the current study are available from the corresponding author upon reasonable request.

## Abbreviations

^18^F-FDG: ^18^F-fluoro-2-deoxy-d-glucose
BSREM: Block sequential regularized expectation maximization
BV: Background variability
CRC_cold_: Cold contrast recovery coefficient
CRC_hot_: Hot contrast recovery coefficient
FWHM: Full width at half maximum
LBS: Lutetium-based scintillator
LE: Residual lung error
LYSO: Lutetium Yttrium Orthosilicate
NEMA IEC: National Electrical Manufacturers Association International Electrotechnical Commission
OSEM: Ordered subset expectation maximization
PET/CT: Positron emission tomography/computed tomography
PMT: Photomultiplier tube
PSF: Point spread function
RDP: Relative difference penalty
ROI: Region of interest
SiPM: Silicon photomultiplier
SNR: Signal-to-noise ratio
SUV: Standardized uptake value
TBR: Target-to-background ratio
